# Adding memory to pressure-sensitive phosphors

**DOI:** 10.1038/s41377-019-0235-x

**Published:** 2019-12-25

**Authors:** Robin R. Petit, Simon E. Michels, Ang Feng, Philippe F. Smet

**Affiliations:** LumiLab, Department of Solid State Sciences, Ghent University, Krijgslaan 281-S1, 9000 Gent, Belgium

**Keywords:** Imaging and sensing, Fluorescence spectroscopy

## Abstract

Mechanoluminescence (ML) is the phenomenon describing the emission of light during mechanical action on a solid, leading to applications such as pressure sensing, damage detection and visualization of stress distributions. In most cases, this mechanical action releases energy that was previously stored in the crystal lattice of the phosphor by means of trapped charge carriers. A drawback is the need to record the ML emission during a pressure event. In this work, we provide a method for adding a memory function to these pressure-sensitive phosphors, allowing an optical readout of the location and intensity of a pressure event in excess of 72 h after the event. This is achieved in the BaSi_2_O_2_N_2_:Eu^2+^ phosphor, where a broad trap depth distribution essential for the process is present. By merging optically stimulated luminescence (OSL), thermoluminescence (TL) and ML measurements, the influence of light, heat and pressure on the trap depth distribution is carefully analysed. This analysis demonstrates that mechanical action can not only lead to direct light emission but also to a reshuffling of trap occupations. This memory effect not only is expected to lead to new pressure sensing applications but also offers an approach to study charge carrier transitions in energy storage phosphors.

## Light: Science & Applications: Sensors: Light-up materials remember when they’ve been touched

Compounds that release light after experiencing mechanical stress can now provide optical readouts of pressure-induced events several days after they occur. Philippe Smet from Ghent University in Belgium and colleagues report that the europium-doped barium silicon oxynitride (BaSi_2_O_2_N_2_:Eu) phosphor exhibits a unique memory function after an initial exposure to ultraviolet light. The phosphor’s excited electrons fill ‘traps’, or defects in the crystal lattice, which can be emptied by applying weight to it, enabling the emission of light. Subsequent illumination of the BaSi_2_O_2_N_2_:Eu crystal with infrared radiation enabled the researchers to release trapped charges in states less accessible to mechanical stimulation. The resulting glow from the phosphor had increased signal strength at locations where pressure actions had previously occurred, even if the pressure action had occurred up to three days before.

## Introduction

When subjecting certain materials to a mechanical action, light emission can be observed. This phenomenon is called mechanoluminescence (ML) and can be induced by many different types of mechanical stress or deformation: friction^1^, fracture^2^, bending^3^, compression^4^, tension^5^, torsion^6^, the impact of a weight^7^, and less common stimulations such as ultrasound^8^, crystallization^9^ and wind^10^. The state of the art in materials and applications has recently been reviewed^9,11,12^. Depending on the specific nature of the mechanical stimulation and whether it results in the destruction of the material, ML is divided into different types and classified as destructive or non-destructive. In the current discussion, ML refers to the emission of light due to the elastic (and thus non-destructive) deformation of the material, also called elastico-mechanoluminescence (EML) or piezoluminescence. This type of ML is particularly interesting for applications since light of relatively high intensity can be generated in a repeatable manner^13–16^. Consequently, ML can be used to indicate stress distributions^17^, microcrack propagation^18^ and structural damages^5,19^ next to a variety of other applications such as displays, the visualization of ultrasound and the mapping of personalized handwriting^9–11,16^. However, the limited range of emission colours, the need for dark conditions, the restriction to real-time measurements and limited signal visibility are currently obstructing the rapid development of ML-based applications^5,20,21^.

Most occurrences of ML in materials are explained by the pressure-induced detrapping mechanism^22–24^, except for some examples, mainly based on ZnS, where no pre-charging is necessary^10,16^. Taking Eu^2+^-doped BaSi_2_O_2_N_2_ phosphor as an example, exciting the phosphor with UV or blue light leads to a 4*f*^7^–4*f*^6^5*d*^1^ electronic transition in Eu^2+^, bringing it into an excited state. The ion can subsequently transit back to the ground state, leading to light emission, with a bluish-green emission colour in this case^25–27^. Alternatively, the excited electron can also be trapped at a defect. Thermal detrapping (and subsequent recombination at the ionized Eu centre) can occur, with the probability depending on the temperature and the energy barrier (or trap depth) related to the trapping defect. This thermally assisted detrapping is the key process in persistent or so-called glow-in-the-dark phosphors^28^, having applications in, e.g., safety signage^29–31^ and bioimaging^32,33^. In addition, the application of pressure can also induce detrapping. As described by the piezoelectrically induced electron detrapping model, the deformation of the material results in the generation of an internal piezoelectric field affecting the electronic structure and thereby the energy barrier^23^. The recombination then leads to the almost immediate emission of light, allowing, for instance, to monitor crack formation in ML phosphor-coated materials in real time^18^. Alternatively, pressure-induced detrapping could also be followed by retrapping at other defects, characterized by a smaller or larger trap depth. Thermal- and pressure-induced detrapping are thus competing processes, and the former (i.e., persistent luminescence, also called afterglow, AG, hereafter) can obscure the latter (i.e., ML).

The possibility for delayed signal acquisition would eliminate most of the current drawbacks of ML-based applications. In this way, one does not have to be physically present during the pressure event to acquire the ML signal, and direct visual contact with the impact area is no longer required. In addition, the presence of background emission, largely consisting of afterglow, can be avoided to increase the visibility of the signal. For this purpose, we introduce the pressure memory (P-MEM) property. This property allows the phosphor particles that were subjected to pressure to be visualized through irradiation with infrared (IR) radiation in excess of 72 h after the application of pressure, allowing us to quantifiably map the applied pressure. In this paper, we investigate the underlying working principle of this P-MEM property. Briefly, it is enabled by the presence of a relatively large range of trap depths in this particular phosphor, with different traps responding in a particular way to specific stimuli (pressure, heat and light). After mechanically induced detrapping, some of the charge carriers recombine, yielding immediate light emission, while the others are redistributed over relatively shallow traps (resulting in a short afterglow) or are almost permanently stored in deep traps. The charges stored in the deep traps can be released by IR radiation, i.e., via optically stimulated luminescence (OSL)^34,35^. In the remainder of this manuscript, the term “ML signal” is used for the combined emission during and shortly after the application of pressure, while the approach of reading out the deep traps via OSL is described by “the P-MEM property”. To be clear, the P-MEM property is to be distinguished from regular OSL, where no mechanical action takes place prior to the optical readout. This work is thus opening new application avenues for pressure sensing. In addition, it also facilitates the study of persistent or energy storage phosphors, as it offers a route to studying trap depth distributions in more detail by probing the subtle interactions between thermal, mechanical and optical detrapping.

## Results

### Reproducibility of ML tests

**Fig. 1:**
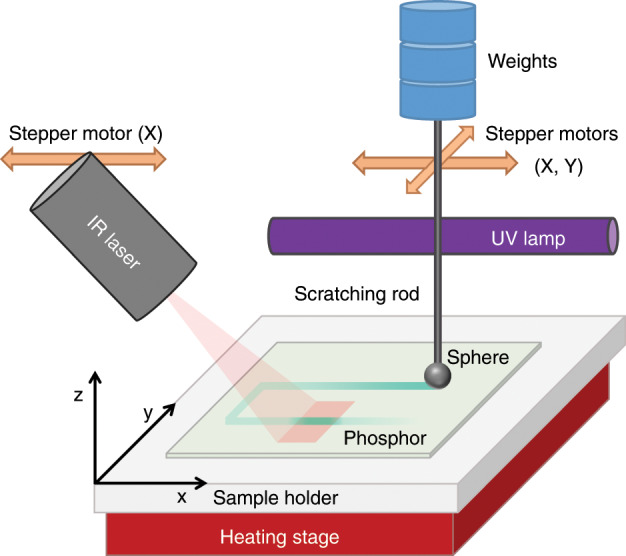
Schematic representation of the experimental setup. It includes a UV excitation source, a motorized friction stage, an IR laser and a digital camera. The camera, omitted to preserve clarity, is mounted at a slight angle to the phosphor–polymer composite sample.

**Fig. 2:**
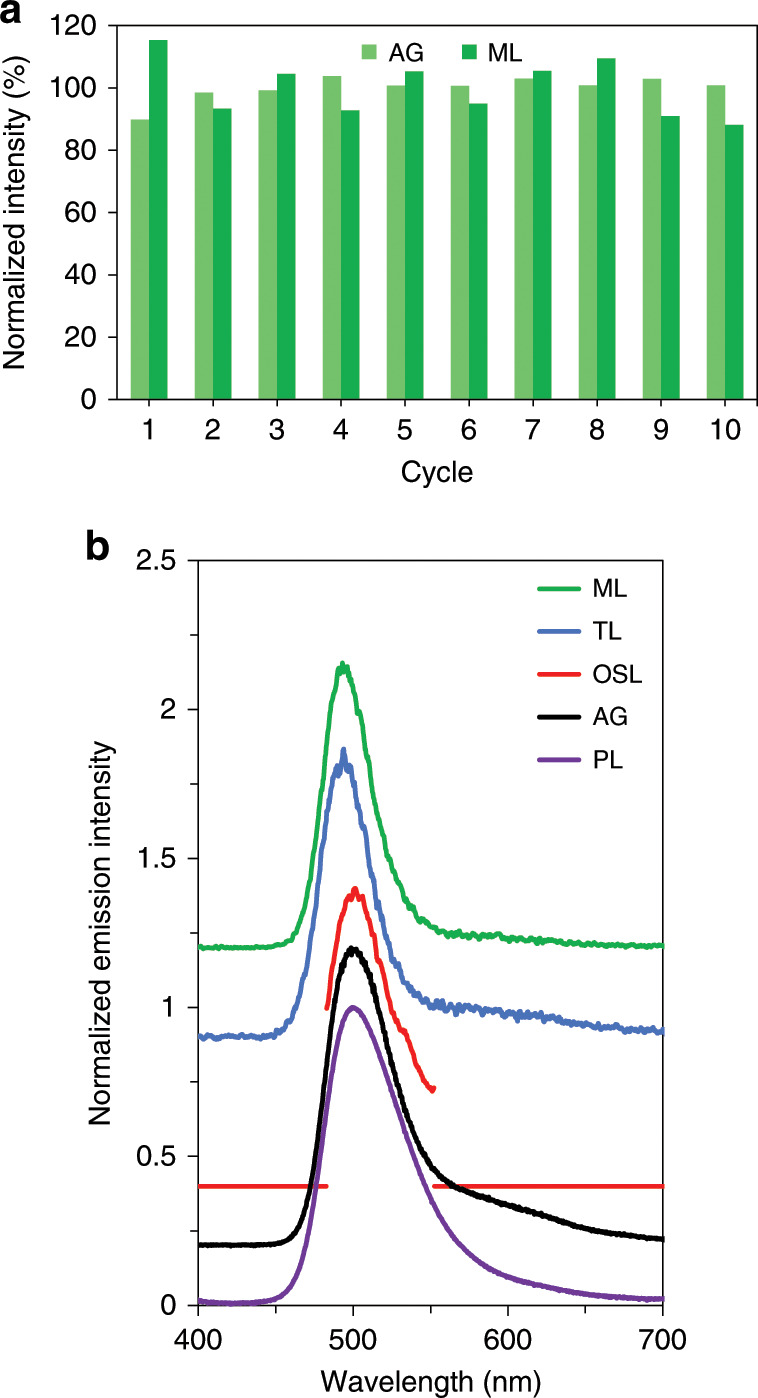
Reproducibility and spectral characterization. **a** Variation of the AG and ML intensity throughout 10 cycles of UV excitation (1 min), waiting (3 min) and dragging the rod over the surface of the phosphor–polymer composite sample. Both AG and ML are normalized to their respective averages. **b** Emission spectra under steady-state excitation (PL), during the afterglow (AG), at the maximum of the thermoluminescence glow peak (TL), during mechanical stimulation (ML) and upon infrared laser irradiation (OSL). To block the reflected IR laser emission, a bandpass filter was used, centred on the emission band for BaSi_2_O_2_N_2_:Eu^2+^.

The mechanical stimulation is performed by dragging a spherical-ended rod over the surface of the phosphor (Fig. 1). The force exerted by the rod on the phosphor surface is tailored by the addition of weights. It is important to confirm that dragging the rod over the sample surface is non-destructive. To demonstrate the reproducibility of the ML measurements as well as the non-destructive nature of the dragging procedure, an experiment consisting of cycles of UV excitation (for 1 min) followed by ML stimulation (3 min after UV excitation) was set up. The reproducibility of the measurements is guaranteed if the initial ML intensity is recovered after each UV excitation step. This ensures that the capacity of the storage traps active in ML remains unaltered by mechanical stimulation. The ML intensity remains essentially constant throughout the repetitions, although a slight variation (*σ* = 9.1%) can be observed (Fig. 2), which is due to the phosphor distribution not being fully homogeneous over the sample surface and slight changes in the precise track followed by the rod during each drag. In addition, the afterglow, which is an indication of the trapping capacity of the phosphor, remains constant (*σ* = 4.0%), indicating the non-destructive nature of the dragging procedure. The ML emission spectrum is essentially identical to the steady-state photoluminescence (PL) emission spectrum and to the spectrum during the afterglow (Fig. 2b).

### P-MEM as enhanced OSL due to mechanical stimulation

**Fig. 3:**
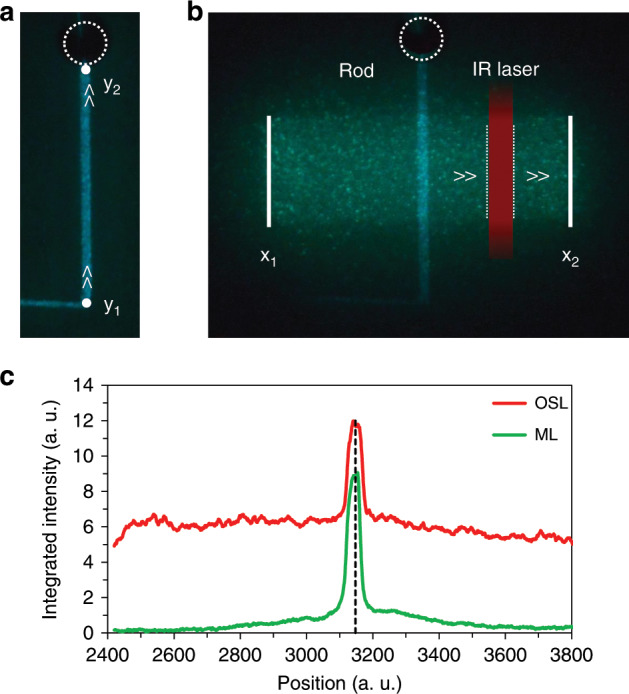
The P-MEM property. **a** After UV excitation and a waiting time of 3 min, the rod was dragged back and forth between positions *y*_1_ and *y*_2_ (approximately 20 mm). Half an hour later, an IR laser was swept from left to right, during which image (**b**) was taken. Finally, the OSL intensity profile (**c**) was calculated within the area confined by *x*_1_ and *x*_2_. For comparison, the ML intensity profile within the same area but measured during the application of pressure is also indicated.

The P-MEM property is achieved through the combination of a mechanical and a subsequent optical stimulation. The phosphor is initially exposed to UV light (365 nm) for 5 min. After a 3 min waiting time to allow the initial afterglow to sufficiently decay, ML is stimulated by dragging the rod back and forth several times. A bright ML emission can be observed (Fig. 3a), which quickly decays after the dragging ends. Half an hour later, the sample is irradiated by the IR laser, which has a vertically elongated beam profile. The laser is swept from left to right at a speed of 2.5 mm s^−1^. A picture is taken by the camera during this sweep (Fig. 3b). Both the integrated ML and OSL profile along the path of the IR laser are shown in Fig. 3c. As can be seen, the P-MEM property emerges as an increase in OSL intensity at the position of the mechanical stimulation. Accordingly, the OSL signal at the position of the mechanical stimulation is thus called the P-MEM signal. It was verified that the emission spectrum during the infrared stimulation originates from the Eu^2+^ luminescent centre in BaSi_2_O_2_N_2_ (Fig. 2b).

**Fig. 4:**
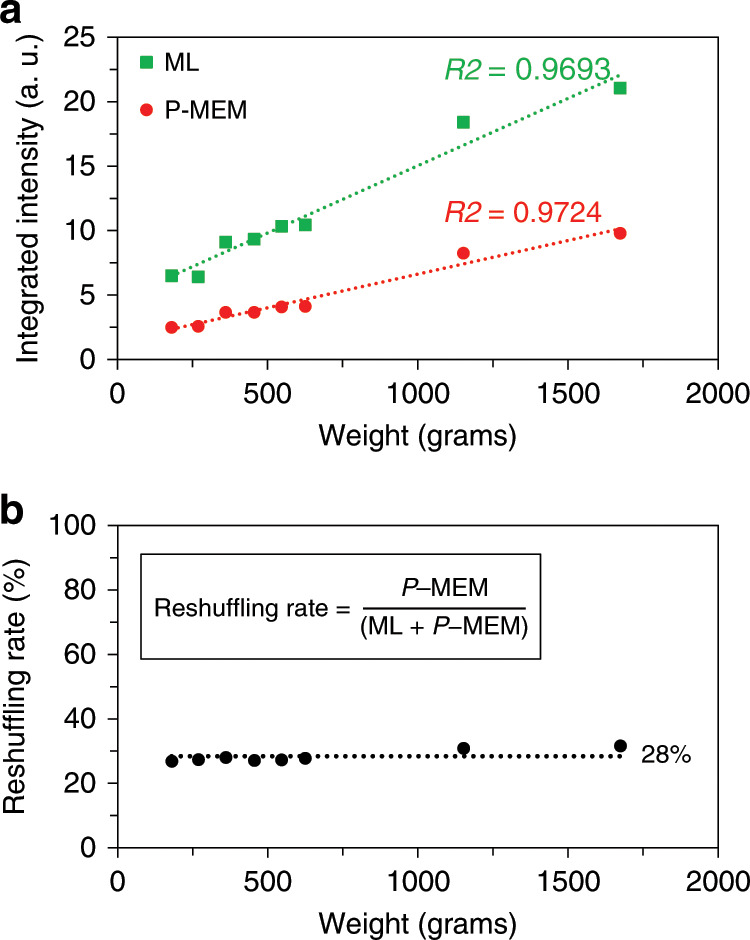
The reshuffling rate: quantifying exchange of trap occupations. **a** Dependency of the ML and P-MEM intensity on the magnitude of the applied load. **b** Ratio of the P-MEM intensity to the total output, consisting of ML and P-MEM, defined as the reshuffling rate.

For most ML phosphors, the ML intensity increases linearly with stress (magnitude of the pressure application) after a threshold^16,36^. The absolute magnitude of the intensity depends on the initial charging, stress amplitude and loading rate. By utilizing this property in ML-based stress sensors, it becomes possible to distinguish between different loadings, provided that the ML intensity is carefully collected and calibrated^37^. The relation between the luminescence intensity and the magnitude of the load was investigated and is shown in Fig. 4a.

As commonly observed for ML, the intensity increases linearly with the applied load. Applying higher loads results in the release of more charge carriers, since more traps are emptied. Some of the released electrons immediately recombine with the ionized europium ions to yield the common ML signal. The remaining fraction is retrapped, with at least some portion going to very shallow traps, yielding a short afterglow after releasing the pressure, as has been observed before for this phosphor^25^. The offset for low pressures upon extrapolating the response curve is presumably related to the fact that the movement of the rod is not completely smooth, leading to significant pressure at lower weights due to a stick-slip motion. By subsequently irradiating the phosphor with IR radiation, a similar linear relation is found between the P-MEM intensity and the applied load (Fig. 4a). The contribution of P-MEM intensity to the total intensity, consisting of ML and P-MEM, is found to adopt a constant value, with an average of 28%, and is represented in Fig. 4b. In this way, since the ML intensity as a function of the applied load follows a linear relation, so does the P-MEM intensity.

**Fig. 5:**
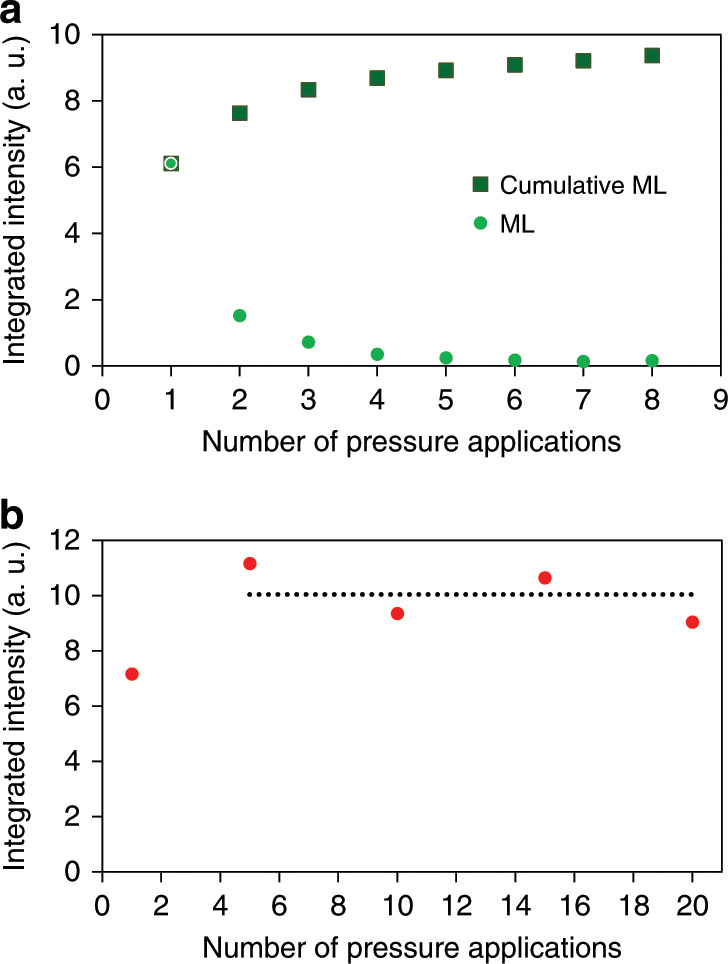
Behaviour of ML and P-MEM intensity under repetitive stimulation. **a** Dependency of the (cumulative) ML and P-MEM intensity on the number of pressure applications. The ML intensity decreases as the number of drags increases, becoming negligible after 6–7 cycles. As a result, the cumulative ML intensity initially increases to the saturation level. **b** Similar to the cumulative ML behaviour in **a**, the P-MEM intensity (indicated by red dots) increases and saturates at a higher number of drags, indicated by the dashed line.

The dependency of the ML and P-MEM intensity on the number of pressure applications is depicted in Fig. 5. After exciting the phosphor, an 8-min window was taken to perform the required number of drags, with the last one aligning with the end of this time window. Then, another 2 min of waiting time were added before starting the IR irradiation to determine the P-MEM intensity. Here, we see that the ML intensity decreases with each additional drag, which is related to the consecutive depletion of traps during each drag. Nevertheless, the cumulative ML intensity shows an initial increase with the number of drags before eventually saturating, with additional drags having no further influence on the observed intensity. This observation can be interpreted as the moment when all traps that can be emptied by a certain pressure have been depleted. The initial increase for the cumulative ML intensity can arise if a singular drag is not capable of emptying all available traps. The subsequent drags then further deplete these trap levels. Eventually, all available traps become empty, causing additional drags to have no further influence on the observed intensity, i.e., saturation of the cumulative ML intensity is achieved. Looking at the P-MEM intensity, we again find a high similarity to the behaviour of the ML intensity, showing an initial increase in intensity followed by a saturation at a higher number of drags (Fig. 5b). These experiments show that the P-MEM intensity follows the behaviour of the (cumulative) ML intensity, which is not unexpected considering that the P-MEM intensity is a fixed percentage of the total number of released charge carriers.

### P-MEM originates from a “reshuffling” of electrons to deep traps

**Fig. 6:**
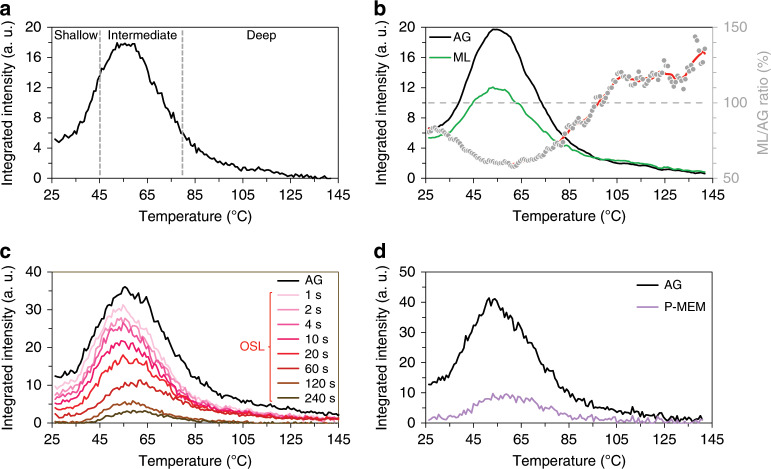
Investigation of the influence of the different stimulations on the occupation of the trap depth distribution. **a** The TL glow curve for BaSi_2_O_2_N_2_:Eu^2+^ is divided into three regions (shallow, intermediate and deep). **b** The TL glow curve obtained after dragging the rod over the surface of the phosphor for 1 min (green line) and the TL glow curve without prior application of pressure (black line). The grey dots show the pointwise ratio of both curves. **c** Influence of the IR irradiation time prior to recording the TL glow curve. **d** The TL glow curve (purple line) after both mechanical (dragging for 1 min) and optical (IR irradiation for 30 s) stimulation compared to the glow curve (black line) without prior stimulation.

Thermoluminescence (TL) is a well-established research tool able to reveal the occupation of traps in phosphors from so-called glow curves, i.e., by observing the light output under linear heating after previous trap filling at lower temperature^38,39^. Generally, the position of a peak in the TL glow curve indicates the trap depth, which is roughly proportional to the corresponding peak temperature, while the (integrated) intensity is a measure of the number of electrons situated on that particular trap. The TL glow curve for BaSi_2_O_2_N_2_:Eu^2+^ reveals that the phosphor contains a broad trap depth distribution (Fig. 6), which is in line with the non-exponential decay of the persistent luminescence intensity^26,40^. The TL emission spectrum is almost identical to the steady-state PL and ML emission spectra (Fig. 2b); hence, this discussion can be limited to evaluating the total TL intensity, integrating the full emission spectrum. It should be noted that for these material systems, recombination at the luminescent centre occurs after the thermally assisted detrapping; hence, no information on the trap depth can be extracted from the shape and position of the TL emission spectrum^39^.

The influence of the different stimulations (dragging, IR irradiation and the combination of both) on the occupation of the trap depth distribution was studied by evaluating the TL glow curves. Each type of stimulation was applied prior to recording the respective TL glow curve. To allow a comparison, a fixed waiting time of 4 min was employed between the end of the charging step by UV excitation and the start of heating for the TL glow curves. The different stimulations were then performed entirely in this 4 min time frame. In this way, the observed TL intensity provides information about which trap levels are influenced by which type of stimulation, upon comparison to the TL intensity in the absence of any stimulation (but still adding the 4-min waiting time), thus corresponding to the initial trap depth distribution. The different TL glow curves are shown in Fig. 6a–d and were divided into three regions: a shallow (25–45 °C), an intermediate (45–80 °C) and a deep part (>80 °C), considering the relative depth of the trap levels. Although the precise temperature ranges are somewhat arbitrary, these ranges correspond to the rapid or slow release of trapped charges (for the shallow and intermediate traps, respectively), whereas the deep traps require strong heating or optical detrapping. In addition, it will become clear that for the specified ranges, there are different responses to the stimuli, such as heat, light or pressure.

Without any stimulation, we can see that the trap depth distribution mainly consists of a large contribution from shallow and intermediate trap levels, while only a small contribution originates from deep trap levels, in agreement with a previous report on this material^26^. Obviously, the delay (4 min) between the excitation of the phosphor and the onset of heating will lower the remaining fraction of electrons at shallow traps due to efficient thermal detrapping. Clearly, mechanical and optical stimulation act differently on the traps present in this compound (Fig. 6a, b). The most dominant effect of mechanical stimulation is the emptying of shallow and intermediate trap levels (Fig. 6b). As the application of pressure leads to charge release (and light emission), fewer traps remain filled at the start of the subsequent heating, yielding a lower intensity when the TL glow curve is recorded. Consequently, the area where the pressure was applied would show up as a negative image, with lower TL compared to non-pressed areas, at least upon heating to no more than 100 °C. In contrast, the occupation of deep traps increased by approximately 20% after mechanical stimulation. This increase in trap occupation is observed more clearly when considering the ratio between the TL glow curves recorded with and without mechanical stimulation, as shown in Fig. 6b. In contrast, the IR irradiation at the studied wavelength of 808 nm initially acts on deep-lying trap levels. This implies the P-MEM property being rooted in a “reshuffling” event in which charge carriers released under the influence of the mechanical stimulation occupy more deep-lying trap levels instead of leading purely to recombination. An increased OSL is then expected to occur where mechanical stimulation had previously occurred because of an increase in electrons in traps highly sensitive to IR stimulation, thus resulting in the P-MEM signal. For increasing duration of the IR irradiation, the emptying of shallow and intermediate trap levels also becomes relevant (Fig. 6c), which can be related to the optical absorption strength of a particular defect at the used wavelength. Moreover, the IR irradiation imparts some heat to the sample when it irradiates a fixed position, as measured by a thermographic camera. The measured increase in surface temperature was 10 °C after 30 s of IR irradiation and then stabilized after an increase of 13 °C after 60 s. Taking this into consideration, the emptying of shallow traps is also partly due to thermally assisted detrapping. For the P-MEM, the laser-induced heating is limited to 2 °C as the laser beam is quickly swept over the phosphor surface; hence, the optical detrapping is the driving force. Compared to the fast depletion of the deep traps upon IR irradiation, the shallow and intermediate traps are governed by a more gradual depletion. Fortunately, the emptying of these traps is not required for obtaining the P-MEM-related signal. If the deep traps would have shown a slow depletion upon IR irradiation, the optical detrapping of the shallow and intermediate traps would overwhelm the signal from the deep traps. Luckily, P-MEM becomes immediately visible as a strong increase in OSL because of the almost instantaneous depletion of a large portion of deep-lying trap levels. Additionally, due to their low density and/or occupancy for a mechanically non-disturbed phosphor, reshuffling of electrons after the application of pressure is more easily noticed. In accordance with the above discussion, combining ML with OSL results in the depletion of shallow and deep traps, as shown in Fig. 6d.

### Visualizing P-MEM

**Fig. 7:**
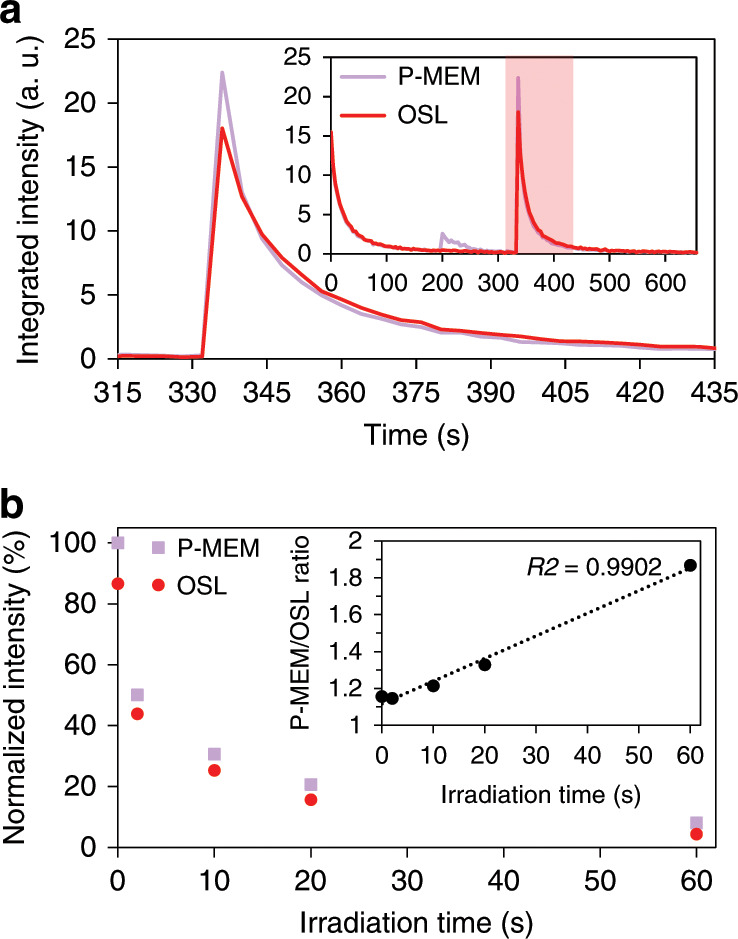
Increasing P-MEM signal visibility. **a** Temporal behaviour of the P-MEM signal. The inset shows the complete duration of the experiment with periods of afterglow (first ~180 s), mechanical stimulation (~180–250 s) and IR irradiation (~330–600 s). The highlighted area is shown in detail in the main figure. **b** Effect of pre-irradiation on the OSL and P-MEM intensity, leading to an increase in contrast between both signals, as shown in the inset.

It is important to stress that one needs to appropriately choose the intensity of the IR irradiation or, equivalently, the speed by which the laser beam is swept over the sample, which will also influence the irradiation intensity. In addition to the P-MEM-related signal, additional optically stimulated detrapping occurs, which obscures the pressure-induced contribution. Therefore, a dedicated experiment was performed to evaluate the influence of the total IR irradiation by keeping the spot of the IR laser at a fixed position. The temporal shape of both the regular OSL (without application of pressure) and the P-MEM (after application of pressure) shows a sharp peak followed by a decay (Fig. 7a). The initial peak is related to the emptying of deep trap levels, while the subsequent decay originates from the gradual depletion of shallow and intermediate trap levels. This is in accordance with the evolution in the shape of the TL glow curves after increasingly longer IR irradiation (Fig. 6c).

Looking at the P-MEM signal as a function of time, we initially see an increase in intensity similar to the one shown in Fig. 3b, where only a short IR irradiation is used. For prolonged IR irradiation (after approximately 10 s, Fig. 7a), the emission intensity adopts lower values compared to the case where no pressure was applied. Two effects are at play. The application of pressure leads to detrapping of the electrons at the shallow and intermediate traps, which partially leads to immediate recombination and partially to trapping at deep traps. As IR irradiation initially acts on the deep traps (Fig. 6c), this leads to an initially increased signal. For longer irradiation times, the shallow and intermediate traps are emptied. In the case of the application of pressure prior to IR irradiation, this will eventually lead to a lower signal because some of the shallow and intermediate traps are depopulated by the mechanical stimuli.

In an attempt to further improve the visibility of the P-MEM signal, the phosphor was briefly irradiated by IR before mechanical stimulation predominantly to empty the deep traps. Although both the OSL signal and the P-MEM signal (without and with the application of pressure prior to the second IR irradiation, respectively) are found to decrease in intensity due to this initial cleaning of the deep traps, the contrast between both signals becomes better for increasing irradiation duration, as shown in Fig. 7b.

**Fig. 8:**
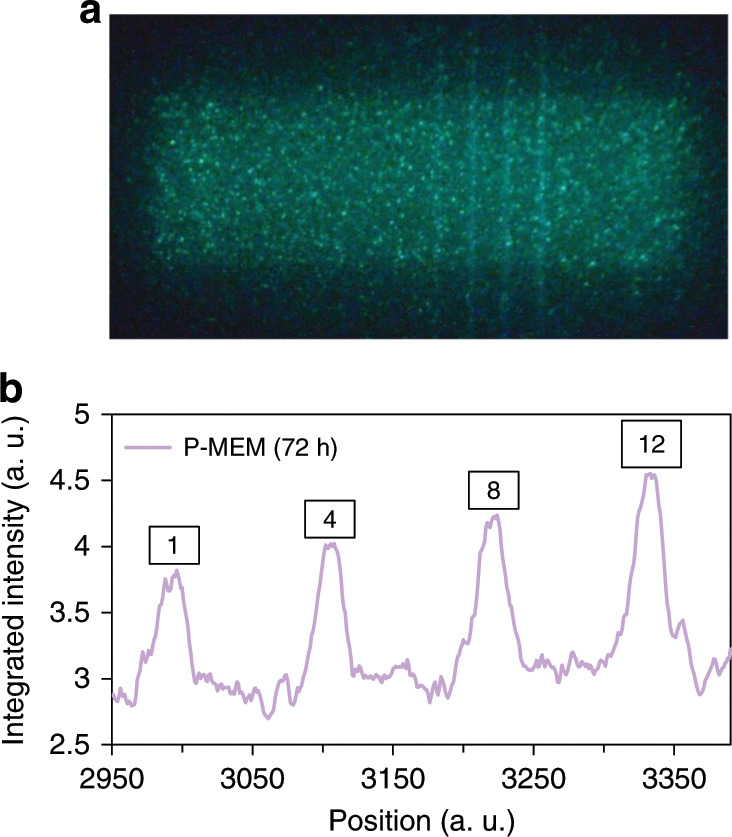
Exploring the limits of the P-MEM property. **a** Digital picture of the sample during irradiation of the phosphor with IR radiation 72 h after mechanical stimulation, consisting of a sequence of drags. **b** Integrated intensity profile derived from **a**, showing the P-MEM intensities corresponding to 1, 4, 8 and 12 drags.

The working principle of P-MEM via reshuffling of trap occupations provides a new way to investigate a pressure event. A merit of this phosphor is that the P-MEM intensity, proportional to the number of electrons being reshuffled from one trap to another, behaves linearly with the magnitude of force over a wide range. The phosphor is characterized by a constant reshuffling rate, defined in Fig. 4b as the ratio between the P-MEM signal (as measured during the IR irradiation) and the combined output of ML (during the application of pressure) and the P-MEM signal. In this way, the phosphor is able to provide pressure sensing with sufficient sensitivity to different loads. The only limit on the signal is the total number of electrons available for reshuffling, determining the attainable magnitude for P-MEM during the readout. Additionally, the visibility of the P-MEM signal is strongly dependent on the background intensity from IR-induced detrapping of shallow and intermediate traps. One way of addressing this is by adopting a sufficiently long delay time between the mechanical stimulation and the OSL readout. Indeed, the P-MEM signal can still be observed with sufficient intensity after a period of 72 h (Fig. 8a, b) between the application of pressure and the readout by IR irradiation.

## Discussion

Deep traps clearly play a crucial role in obtaining P-MEM. Due to their long average lifetimes, it is expected that the occupation at deep traps, being sensitive to the application of pressure, does not change significantly as long as the temperature remains close to room temperature. However, the same does not hold true for the shallow and intermediate traps, which can be thermally emptied over the course of minutes to days. As shown in Fig. 8, the P-MEM signal is still observable after a waiting time of 3 days between the mechanical stimulation and the IR readout. The added value of waiting this long is that the background signal due to shallow and intermediate traps is greatly decreased, increasing the visibility of P-MEM. Taking this into consideration, the delay can possibly be extended to even longer times for BaSi_2_O_2_N_2_:Eu^2+^ and other phosphors, depending on their trap depth distribution and storage capacity.

Clearly, this opens a new opportunity for information storage and retrieval. The mechanical stimulation provides a unique way to write information, in addition to the methods of writing by photoexcitation^41,42^. This information can be stored in the phosphor for an increased period of time because the reshuffling of electrons to deep traps minimizes the thermal detrapping at ambient temperature. The readout by OSL, compared to a thermal readout, can provide not only the magnitude of force but also its spatial distribution, with the possibility of improving the contrast by adopting long waiting times or an initial cleaning step. Therefore, P-MEM has great potential in areas such as structural health monitoring, where the determination of these properties is of utmost importance^2,5,9,37,43^. It is noted that the reshuffling of electrons over the available electron traps and the faster responsivity of deep traps to IR are important for the P-MEM signal to be clearly visible on top of the regular OSL. A small amount of reshuffling would not bring about a significant increase in the number of electrons at deep traps. These factors can be utilized to search for other potential P-MEM materials. Considering that many ML phosphors have broad trap depth distributions, finding suitable phosphors in addition to BaSi_2_O_2_N_2_:Eu^2+^ is very likely.

In contrast to the mechanically induced reshuffling in BaSi_2_O_2_N_2_:Eu^2+^, the combination of trap depth distribution and reshuffling by means of non-mechanical stimuli has been reported in other phosphors, e.g., in the case of persistent phosphors for bioimaging. In vivo optical imaging for biomedical applications requires red or near-infrared (NIR) emitting persistent luminescent materials to reduce absorption and scattering in the biological tissue and avoid the large background due to autofluorescence upon excitation. Achieving high imaging resolution and deep tissue penetration from phosphors with intense and long-duration persistent luminescence in excess of 1000 nm is still an issue. Recently, a reshuffling or transfer mechanism was reported for ZnGa_2_O_4_ doped with Cr^3+^, LiGa_5_O_8_ doped with Cr^3+^ and Y_3_Al_2_Ga_3_O_12_ codoped with Er^3+^ and Cr^3+^, opening the possibility for excitation-free and background-free optical imaging^42,44,45^. In all three compounds, the reshuffling of charges from deep traps to shallow traps is realized by photostimulation (977 nm NIR lamp, white LED, 660 nm LED), observed in the TL glow curves as an increase in occupation at shallow trap levels. The subsequent thermal release then results in an increased persistent luminescence. By depleting the deep traps using photostimulation pulses, persistent luminescence can be perceived over a much longer time without any background due to excitation. Similar to P-MEM providing a solution for the fast decay of ML, this reshuffling overcomes the quick persistent luminescent fading limiting the duration of optical imaging. Often resulting in counter-intuitive properties at first, the added value these reshuffling mechanisms bring to existing applications definitely make them worth exploring, potentially leading to the development of novel applications making full use of the trap depth distribution present in the phosphor. They demonstrate that we still know relatively little about the inner workings of the luminescent phenomena in terms of detrapping and retrapping routes in addition to the nature and interaction of the traps involved, warranting further in-depth research.

In conclusion, we have reported a specific interaction between mechanical and optical detrapping in BaSi_2_O_2_N_2_:Eu^2+^, leading to the so-called P-MEM property. This interaction allows a pressure-induced ML signal to be recovered through irradiation of the phosphor with IR radiation. The origin of the occurrence of the P-MEM property was investigated through TL glow curves. Careful analysis revealed that, in addition to ML, mechanical stimulation leads to the reshuffling of charge carriers towards deep-lying traps. Upon optical detrapping by means of IR irradiation, these deeper traps are rapidly and predominantly emptied, leading to an increased signal strength at places where a pressure action had previously occurred. A delay of 72 h between the pressure stimuli and the readout could be achieved. The signal intensity during the optical readout is directly proportional to the direct light emission during the pressure application. Future investigations concentrating on improving the P-MEM signal, e.g., by variation of the wavelength for optical detrapping or by trap depth engineering, as well as exploring the P-MEM property in other phosphors containing a broad trap depth distribution, are envisioned to lower the threshold for the development of applications based on this phenomenon.

## Materials and methods

### Sample preparation

All measurements shown in this paper were executed on a single phosphor, namely, BaSi_2_O_2_N_2_:Eu^2+^. This phosphor was synthesized using a high-temperature solid-state reaction. For this, stoichiometric amounts of starting materials BaCO_3_ (99.95%, Alfa Aesar) and Si_3_N_4_ (α-phase, 99.5%, Alfa Aesar) were weighed and mixed with each other after thoroughly grinding them in an agate mortar. Appropriate amounts of BaCO_3_ were substituted with EuF_3_ (99.5%, Alfa Aesar) to substitute 2% of the Ba ions with Eu. This mixture was then placed in a horizontal-tube oven at a temperature of 1425 °C for a period of 4 h. To obtain predominantly Eu^2+^ ions, a reducing atmosphere was introduced (90% N_2_, 10% H_2_). The crystal structure of the as-synthesized phosphor powder was checked by *θ*−2*θ* X-ray diffraction measurements and compared with literature data. These results showed the structure of the BaSi_2_O_2_N_2_ host compound, as could be expected from the low dopant concentrations of Eu^25^. Finally, a phosphor–polymer composite sample was prepared by embedding the phosphor powder in a transparent polymer binder material (EpoFix, Struers GmbH). Typical dimensions are 38 mm (length) by 40 mm (width) by 2 mm (height).

### Experimental setup

A setup was built allowing the sequential stimulation of ML, OSL and TL. This setup contains a UV excitation source (365 nm, phosphor converted mercury vapour fluorescent lamp, 6 W), a motorized friction stage to stimulate the ML (8MT173-20-240 mA, range of 20 mm, Standa stepper motors), an IR laser to induce the OSL (RLDB808-120-3, Dot Laser Module-808 nm, 120 mW, 40 mm^2^ spot size, Roithner) and a digital camera to detect the emitted light (Nikon DSLR DX D3200). The friction stage consists of a metal rod with a ball-shaped end (diameter: 4 mm) to which a specific mass can be attached. The rod is positioned via the motorized stage in the lateral (*x*, *y*) directions, while the rod presses down on the sample surface in the perpendicular (*z*) direction. To minimize the influence of environmental light as well as the IR radiation emitted by the laser, measurements were executed in a dark environment, and the camera was equipped with a bandpass filter (520 nm CWL, 50 mm diameter, 70 nm bandwidth, OD 6 fluorescence filter, Edmund Optics). For the TL measurements, a heating stage was added to the setup, consisting of heating resistors mounted underneath the sample holder. The heating rate achieved by increasing the current (100 mA min^−1^) was found to be linear and amounted to 10 °C min^−1^, measured using a thermographic camera (A35sc, FLIR systems).

### Detection and analysis

The luminescence of the phosphor was captured by the digital camera either as a series of pictures separated from each other by a predetermined time interval or as individual pictures taken at the desired times. These pictures were processed by self-written software routines (MATLAB), combining the appropriate colour channels and subtracting the background and camera noise, resulting in net luminescence intensities. The ML, OSL, P-MEM and TL intensities were derived by integrating the associated areas in the pictures. It is important to note that the ML and OSL intensities were obtained from pictures with a sufficiently long exposure time (10 s) to capture the pressure event and the readout by IR irradiation, respectively. For ML, this means that the intensity corresponds to the amount of light emitted while dragging the rod over the surface of the phosphor (immediate ML and the initial afterglow). Similarly, the OSL intensity corresponds to the light emitted during the irradiation of the phosphor using the IR laser, which performs a scanning motion from left to right. Emission spectra (PL, AG, TL, ML and OSL) were recorded using a ProEM1600 EMCCD camera attached to an Acton SP2300 monochromator (Princeton Instruments). An optical fibre mounted in close proximity to the phosphor surface was used to collect the light. To block the reflected IR laser emission, a bandpass filter (Edmund Optics) was used.
